# Supplementary material for: Empirical methods for controlling false positives and estimating confidence in ChIP-Seq peaks
Source: BMC Bioinformatics. 2008 Dec 5;9:523. doi: 10.1186/1471-2105-9-523 (PMC2628906; doi:10.1186/1471-2105-9-523)
Supplement: Additional File 1 — A variety of html documents from the USeq web site detailing the available applications, their best usage, output file type descriptions, command line menus, etc. [file 1471-2105-9-523-S1.zip › USeqUserGuides/JavaDoc.zip!/JavaDoc/edu/utah/seq/data/ComparatorInfoPoint.html]

ComparatorInfoPoint


|  |  |  |
| --- | --- | --- |
| **Overview** \| **Package** \| **Class** \| **Use** \| **Tree** \| **Deprecated** \| **Index** \| **Help** | |  |
| **PREV CLASS**   **NEXT CLASS** | **FRAMES**    **NO FRAMES**     **All Classes** |  |
| SUMMARY: NESTED \| FIELD \| CONSTR \| METHOD | DETAIL: FIELD \| CONSTR \| METHOD |  |


---


## edu.utah.seq.data Class ComparatorInfoPoint

```
java.lang.Object
  edu.utah.seq.data.ComparatorInfoPoint
```

**All Implemented Interfaces:**: java.util.Comparator

---

``` public class ComparatorInfoPoint extends java.lang.Object implements java.util.Comparator ```

Class to sort Info by chromosome.

---

**Constructor Summary**

`ComparatorInfoPoint()`


| **Method Summary** | |
| --- | --- |
| `int` | `compare(java.lang.Object arg0, java.lang.Object arg1)`             Sorts by name |

**Methods inherited from class java.lang.Object**

`equals, getClass, hashCode, notify, notifyAll, toString, wait, wait, wait`

**Methods inherited from interface java.util.Comparator**

`equals`

**Constructor Detail**

### ComparatorInfoPoint

```
public ComparatorInfoPoint()
```


**Method Detail**

### compare

```
public int compare(java.lang.Object arg0,
                   java.lang.Object arg1)
```

:   Sorts by name

    :   **Specified by:**: `compare` in interface `java.util.Comparator`


---


|  |  |  |
| --- | --- | --- |
| **Overview** \| **Package** \| **Class** \| **Use** \| **Tree** \| **Deprecated** \| **Index** \| **Help** | |  |
| **PREV CLASS**   **NEXT CLASS** | **FRAMES**    **NO FRAMES**     **All Classes** |  |
| SUMMARY: NESTED \| FIELD \| CONSTR \| METHOD | DETAIL: FIELD \| CONSTR \| METHOD |  |


---
